# A Machine Learning Approach for the Differential Diagnosis of Alzheimer and Vascular Dementia Fed by MRI Selected Features

**DOI:** 10.3389/fninf.2020.00025

**Published:** 2020-06-11

**Authors:** Gloria Castellazzi, Maria Giovanna Cuzzoni, Matteo Cotta Ramusino, Daniele Martinelli, Federica Denaro, Antonio Ricciardi, Paolo Vitali, Nicoletta Anzalone, Sara Bernini, Fulvia Palesi, Elena Sinforiani, Alfredo Costa, Giuseppe Micieli, Egidio D'Angelo, Giovanni Magenes, Claudia A. M. Gandini Wheeler-Kingshott

**Affiliations:** ^1^NMR Research Unit, Department of Neuroinflammation, Faculty of Brain Sciences, Queen Square MS Centre, UCL Queen Square Institute of Neurology, London, United Kingdom; ^2^Department of Electrical, Computer and Biomedical Engineering, University of Pavia, Pavia, Italy; ^3^Brain MRI 3T Research Center, IRCCS Mondino Foundation, Pavia, Italy; ^4^Stroke Unit, IRCCS Mondino Foundation, Pavia, Italy; ^5^Laboratory of Neuropsychology and Unit of Behavioral Neurology, IRCCS Mondino Foundation, Pavia, Italy; ^6^Department of Brain and Behavioral Sciences, University of Pavia, Pavia, Italy; ^7^Headache Center, IRCCS Mondino Foundation, Pavia, Italy; ^8^Department of Medical Physics and Biomedical Engineering, Centre for Medical Image Computing, University College London, London, United Kingdom; ^9^Radiology Unit, IRCCS Policlinico San Donato, Milan, Italy; ^10^Scientific Institute H.S. Raffaele Vita e Salute University, Milan, Italy; ^11^Department of Emergency Neurology, IRCCS Mondino Foundation, Pavia, Italy; ^12^Brain Connectivity Center, IRCCS Mondino Foundation, Pavia, Italy

**Keywords:** Alzheimer disease, vascular dementia, machine learning, resting state fMRI, DTI

## Abstract

Among dementia-like diseases, Alzheimer disease (AD) and vascular dementia (VD) are two of the most frequent. AD and VD may share multiple neurological symptoms that may lead to controversial diagnoses when using conventional clinical and MRI criteria. Therefore, other approaches are needed to overcome this issue. Machine learning (ML) combined with magnetic resonance imaging (MRI) has been shown to improve the diagnostic accuracy of several neurodegenerative diseases, including dementia. To this end, in this study, we investigated, first, whether different kinds of ML algorithms, combined with advanced MRI features, could be supportive in classifying VD from AD and, second, whether the developed approach might help in predicting the prevalent disease in subjects with an unclear profile of AD or VD. Three ML categories of algorithms were tested: artificial neural network (ANN), support vector machine (SVM), and adaptive neuro-fuzzy inference system (ANFIS). Multiple regional metrics from resting-state fMRI (rs-fMRI) and diffusion tensor imaging (DTI) of 60 subjects (33 AD, 27 VD) were used as input features to train the algorithms and find the best feature pattern to classify VD from AD. We then used the identified VD–AD discriminant feature pattern as input for the most performant ML algorithm to predict the disease prevalence in 15 dementia patients with a “mixed VD–AD dementia” (MXD) clinical profile using their baseline MRI data. ML predictions were compared with the diagnosis evidence from a 3-year clinical follow-up. ANFIS emerged as the most efficient algorithm in discriminating AD from VD, reaching a classification accuracy greater than 84% using a small feature pattern. Moreover, ANFIS showed improved classification accuracy when trained with a multimodal input feature data set (e.g., DTI + rs-fMRI metrics) rather than a unimodal feature data set. When applying the best discriminant pattern to the MXD group, ANFIS achieved a correct prediction rate of 77.33%. Overall, results showed that our approach has a high discriminant power to classify AD and VD profiles. Moreover, the same approach also showed potential in predicting earlier the prevalent underlying disease in dementia patients whose clinical profile is uncertain between AD and VD, therefore suggesting its usefulness in supporting physicians' diagnostic evaluations.

## Introduction

Alzheimer disease (AD) is the primary and most frequently diagnosed dementia disease in elderly subjects. At a physiological level, AD is a progressive neurodegenerative disease characterized by the accumulation of amyloid-β plaques and tau-related neurofibrillary tangles mainly affecting the prefrontal and mesial-temporal areas of the brain. AD is associated with memory dysfunction and severe cognitive decline caused by a dramatic shrinking of the brain tissues (i.e., atrophy) and neural circuitries (Reitz et al., 2011; Serrano-Pozo et al., 2011). The accurate diagnosis of AD is crucial for patients' management and treatment, but it is often challenging, in particular when AD-like symptoms overlap with cerebrovascular changes, which are also a characteristic trait of vascular dementia (VD) (Groves et al., 2000). From an epidemiological point of view, VD is considered the second most prevalent type of dementia after AD. VD represents a clinical syndrome that includes a wide spectrum of cognitive dysfunctions resulting from brain tissue damage caused by vascular disease that can lead to large artery strokes, small vessel disease (SVD), and other less-frequent vascular lesions (Micieli, 2006; Vinters et al., 2018). From a clinical point of view, VD also represents a great challenge because of its relatively high prevalence and lack of effective treatment options (Baskys and Hou, 2007). Indeed, although cognitive impairment following stroke generally tends to recede, vascular dementia due to SVD is often progressive and may be confused with AD, possibly leading to delays and errors in identifying the best treatment for each individual.

A relevant help in characterizing dementia has come from advanced magnetic resonance imaging (MRI) techniques, such as diffusion tensor imaging (DTI) and resting-state functional magnetic resonance imaging (rs-fMRI) (Agosta et al., 2017; Filippi et al., 2019). A recent DTI study has shown that AD and VD are characterized by distinct patterns of white matter (WM) changes, therefore suggesting DTI parameters as valid biomarkers to investigate the pathogenesis of dementia (Palesi et al., 2018). Several studies have instead used rs-fMRI to investigate the brain functional connectivity (FC) changes caused by neurodegeneration, providing important insights into the pathophysiology of dementia (Castellazzi et al., 2014; Buckley et al., 2017) as well as into the mechanism of disease progression (Dillen et al., 2017). However, despite the large number of MRI studies focused on dementia, the identification of MRI biomarkers to clearly differentiate the AD profile from VD remains difficult.

Improvements in imaging and the advent of affordable powerful computational resources have created a fertile ground for the development of machine learning (ML) approaches, which represent a pool of qualified methods for exploring data to discover already present unknown patterns (Bishop, 2006). Indeed, ML techniques, combined with MRI-derived indices, i.e., quantitative MRI (qMRI), have been used to identify AD subjects from normal elderly people (Long et al., 2017). Other studies have shown that the combination of ML with qMRI represents a suitable approach not only to automatically identify dementia diseases, but also to predict the disease progression as well as the conversion from a mild cognitive impairment (MCI) to a more severe condition, such as AD (Dyrba et al., 2015; Dallora et al., 2017; Pellegrini et al., 2018). Moreover, a recent study showed that ML combined with volumetric measurements derived from structural MRI represents a useful approach for the differential diagnosis between AD and VD (Zheng et al., 2019).

Compared to earlier pieces of work, this study aims to establish the potential of ML algorithms combined with advanced qMRI metrics to automatically discriminate AD from VD. Moreover, we evaluate which algorithms are more suitable in enhancing classification accuracy when using multimodal MR features rather than unimodal data. Finally, we test whether this approach is able to give an earlier and more precise indication (compared to conventional clinical evaluations) about the prevalent underlying disease (i.e., AD rather than VD) in a pool of patients diagnosed with a “mixed” VD–AD dementia (MXD) profile.

## Materials and Methods

### Subjects

MRI acquisitions were performed on a total data set of 77 subjects with dementia. Thirty-three subjects diagnosed with AD (age 72.8 ± 7.3), and 27 subjects diagnosed with VD (age 76.6 ± 7.7) were recruited for the study. A third group of 15 subjects diagnosed with mixed AD–VD dementia (MXD, age 76.3 ± 6.7) was included to test the potential of the proposed ML approach in predicting the prevalent underlying dementia disease. AD, VD, and MXD patients were selected on the basis of the NINCDS2-ARDA criteria (McKhann et al., 2011) among those regularly attending the Neurological Institute IRCCS Mondino Foundation (Pavia, Italy). Exclusion criteria were age >80 years and significant medical or neurological (other than AD or VD) or psychiatric disease. Patients with significant central nervous system (CNS) disorders (e.g., Parkinson's disease and other extra-pyramidal disorders, multiple sclerosis, epilepsy, clinical evidence of acute ischemic or hemorrhagic stroke, known intracranial lesions, systemic causes of subacute cognitive impairment, and/or previous head injury with loss of consciousness) were excluded, too. All subjects were scanned under an institutional review board (IRB) approved protocol after obtaining written informed consent from them or their lawful caregiver.

### Clinical and Neuropsychological Assessment

All subjects underwent clinical and neuropsychological evaluation to assess their global cognitive status using the Mini-Mental State Examination (MMSE) (Folstein et al., 1975) and the following cognitive domains: attention (Stroop test, trail making test A and B, attentive matrices), memory (digit and verbal span, Corsi block-tapping test, logical memory, Rey–Osterrieth complex figure recall, Rey 15 item test), language (phonological and semantic verbal fluency), executive function (Raven's matrices, Wisconsin card sorting test, frontal assessment battery), visuospatial skills (Rey-Osterrieth complex figure) (Carlesimo et al., 1996; Bianchi and Dai Prà, 2008). Raw scores were corrected for age and education and then transformed into equivalent scores, ranging from zero (pathological) to four (normal). For each cognitive domain, a weighted score was obtained averaging the value of the equivalent scores of all tests belonging to that specific cognitive domain (van Dijk et al., 2008). Clinical classification of AD or VD was performed according to the abovementioned criteria and was further refined by excluding patients with mixed dementia according to the Hachinski scale (HS) with pathology-validated cutoffs (Hachinski et al., 1975; Moroney et al., 1997): pure VD (HS ≥ 7), pure AD (HS ≤ 4), and MXD (HS 5 and 6). Vascular alterations were semi-quantitatively rated on radiological bases by evaluating white matter (WM) leukoaraiosis using the Fazekas scale (Fazekas et al., 1987).

### MRI Acquisitions

All subjects underwent MRI examination using a 3T Siemens Skyra scanner (Siemens, Erlangen, Germany) with a 32-channel head coil. The MRI acquisition protocol included (1) rs-fMRI: T2^*^-weighted gradient echo echo-planar (GRE-EPI) sequence (TR/TE = 3,010/20 ms; voxel size = 2.5 mm isotropic, FOV = 224 mm, 60 slices, 120 volumes) and (2) DTI: twice refocused spin echo echo-planar (SE-EPI) sequence (TR/TE = 10,000/97 ms, 70 slices with no gap, acquisition matrix = 120 × 120, voxel size = 2 mm isotropic, 64 diffusion-weighted directions, b = 1,200 s/mm^2^, 10 volumes with no diffusion weighting). A high-resolution 3-D sagittal T1-weighted (3-D T1) scan (MPRAGE sequence: TR/TE/TI = 2,300/2.95/900 ms, flip angle 9°, 256 slices, voxel size = 1 × 1 × 1.2 mm^3^, FOV = 270 mm) was also acquired for anatomical reference.

### Image Processing and Data Analysis

Image analysis was carried out using the FSL tools (FMRIB Software Library, version 5.0.9, http://www.fmrib.ox.ac.uk/fsl/) and Matlab (v. R2018b, The Mathworks, Inc., Natick, MA).

### DTI Analysis

#### Data Preprocessing

First, for each subject, the 10 volumes acquired with no diffusion weighting (b0 = 0 s/mm^2^) were averaged to obtain a mean b0 volume. DTI volumes were then corrected for motion and eddy current distortions using the eddy tool, which aligns the diffusion-weighted volumes to the mean b0 image. A binary brain mask was obtained from the mean b0 volume using the brain extraction tool (BET). DTIFIT was used to generate individual fractional anisotropy (FA) and mean diffusivity (MD) maps. For each subject, the 3-D T1 images were first segmented with the FAST algorithm of FSL to produce the WM and GM maps (as well as whole brain by their addition). The FA map was then aligned to the respective 3-D T1 volume using a full-affine registration with a windowed-sinc interpolation (using a Hanning window of size 7 × 7 × 7) using the FMRIB's linear image registration tool (FLIRT). The mean FA value of the brain was obtained by overlapping the brain mask with the aligned FA map.

#### Tract-Based Spatial Statistics

Tract-based spatial statistics (TBSS) was performed on DTI images to investigate the voxel-wise distribution of FA and MD differences among groups. This analysis was carried out using the TBSS tool as implemented in FSL and following the pipeline reported in Smith et al. (2006). The FA maps of all the subjects were aligned to the best target and then to a common space (MNI152 space) by non-linear registration and averaged to obtain a mean FA skeleton. Finally, each subject's aligned FA data were projected onto the mean FA skeleton. MD maps were also projected into the mean FA skeleton using the same projector vectors that were obtained in the FA maps alignment. A GLM was applied to assess differences in FA and MD between patients and HC. The results of this study have been fully reported in Palesi et al. (2018).

#### Features Extraction From DTI

**Figure 1:**
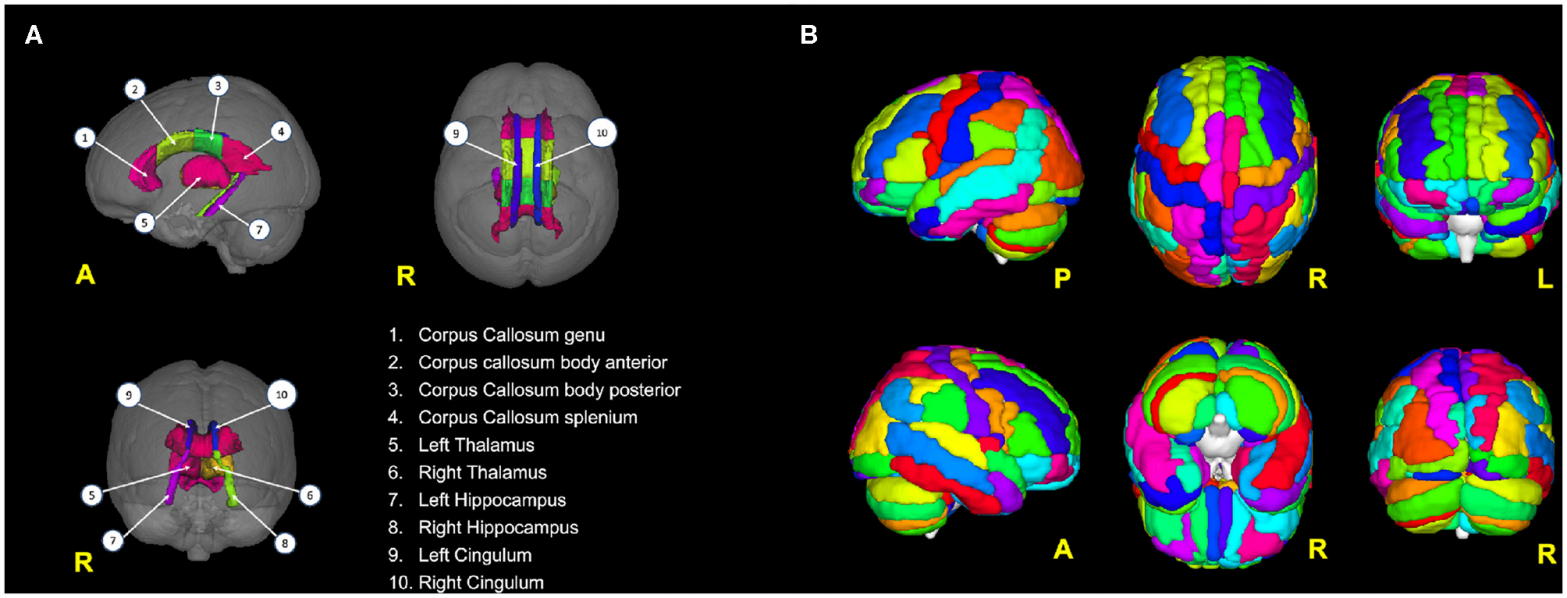
**(A)** Brain areas from which DTI features (FA, MD) have been extracted; **(B)** the 116 areas from the AAL atlas used to parcellate the brain and then to calculate the rs-fMRI-derived graph theory (GT) metrics.

The areas that resulted in being particularly relevant from the TBSS analysis were saved as regions of interest (ROIs, see Figure 1A and Table 1). For each ROI, we then extracted mean FA and MD values. These extracted values were used as DTI-derived features for the ML approach of this study.

**Table 1:** Overview of the imaging features used to form the three data sets: DTI, fMRI GT, and DT + fMRI GT data sets.

**Dataset**	**ROI**	**Imaging features**	**N features**
DTI	Corpus callosum (genu, body anterior, body posterior, splenium) thalamus (left, right) hippocampus (left, right) cingulum (left, right)	FA, MD	20
fMRI GT	116 AAL areas	Nodal degree (DEG) participation coefficient (PC) betweenness centrality (BC) clustering coefficient (CC) normalized local efficiency (Eloc_norm_) global efficiency (Eglob) graph average CC (Cm)	698
DTI + fMRI GT	Corpus callosum (genu, body anterior, body posterior, splenium) thalamus (left, right) hippocampus (left, right) cingulum (left, right) 116 AAL areas	FA, MD nodal degree (DEG) participation coefficient (PC) betweenness centrality (BC) clustering coefficient (CC) normalized local efficiency (Eloc_norm_) global efficiency (Eglob) graph average CC (Cm)	718

### rs-fMRI Analysis

#### Data Preprocessing

Individual subject's preprocessing consisted of motion correction, brain extraction, spatial smoothing using a Gaussian kernel of full-width-at-half-maximum (FWHM) of 5 mm, and high-pass temporal filtering equivalent to 150 s (0.007 Hz). For each subject, rs-fMRI volumes were then registered to the corresponding structural 3-D T1w scan using the FMRIB's linear image registration tool (FLIRT) and subsequently to standard space (MNI152) using FMRIB's non-linear image registration tool (FNIRT) with default options. Moreover, to reduce the nuisance effects of non-neuronal BOLD fluctuations, white matter (WM) and cerebrospinal fluid (CSF) signals were regressed out from rs-fMRI data.

#### Brain Network Computation

For each subject, preprocessed rs-fMRI images were parcellated using the automated anatomical labeling (AAL) atlas into 116 distinct areas (Tzourio-Mazoyer et al., 2002) that defined the *nodes* of the brain network (Figure 1B; see also Table S1). For each AAL area, the mean rs-fMRI signal was calculated by averaging the time series of all the voxels within the AAL region. The *edges* of the brain network were defined as the functional connectivity of all pairs of 116 AAL areas using Pearson's correlation coefficient. This operation generated for each subject a weighted undirected functional connectivity matrix, which corresponded to a dense network. Each subject-specific connectivity matrix was then thresholded by preserving a proportion *P* (0 < *P* < 1) of the strong weights, which corresponds to the number of the retained strong weights divided by the total number of weights. All diagonal weights (self-connections) were set to zero. *P* was set at 12% to obtain the optimal matrix sparsity as suggested by Rubinov and Sporns (2010).

#### Graph Metrics Computation

Each graph (i.e., thresholded correlation matrix) was then treated in Matlab with the Brain Connectivity Toolbox (BCT) to compute different metrics from graph theory (GT) to investigate the properties of the 116 AAL brain regions. Specifically, three measures of functional segregation: the clustering coefficient (CC), the graph average CC (Cm), and the normalized local efficiency (Eloc_norm_) were calculated to characterize the ability of the brain for specialized processing to occur within a densely interconnected group of regions. A functional integration measure, global efficiency (Eglob), was used to assess the ability of the brain to rapidly combine specialized information from distributed regions. Moreover, three local nodal measures were calculated: nodal degree (DEG), participation coefficient (PC), and betweenness centrality (BC) (Rubinov and Sporns, 2010).

### Data Sets for Machine Learning

All MRI features were obtained from the baseline visit of the patients. For each subject, the measures extracted from DTI (i.e., mean FA and MD values from 10 selected brain areas) and rs-fMRI (graph metrics from 116 AAL areas) were combined in a vector of parameters (i.e., a record). The records of all subjects were then collected to create the following data sets (see also Table 1):

1. DTI data set (unimodal data set): This contained only the DTI-derived metrics (20 features per subject). Therefore, it was considered a unimodal data set.
2. GT data set (unimodal data set): This contains the 698 graph theoretical metrics per subject, derived from rs-fMRI images. This was also considered a unimodal data set.
3. DTI + GT data set (multimodal data set): This was obtained unifying (1) and (2) into a single data set, which resulted in 718 features per subject.

Each data set was screened to remove outliers by deleting those records that contained more than 30% of features laying three standard deviations (*SD*) away from the sample's mean. The threshold of 3 *SD* was chosen as a good value to remove the spurious feature values, which definitively fell outside the 99.7% of their distributions, from the database (Rousseeuw and Hubert, 2011). Different thresholding percentages on the subjects' features were tested, varying between 10 and 80%, and the 30% threshold resulted in the best compromise between data cleaning and data preservation. Moreover, for each data set, data that survived the outlier-removal procedure were standardized (i.e., *z*-score normalized) prior to beginning the following steps of the analysis. In order to check whether multimodal features were superior to unimodal ones in separating the two patients' groups, each ML algorithm was run separately on three distinct data sets: DTI data set (unimodal), GT fMRI data set (unimodal), and DTI + fMRI GT data set (multimodal).

### Feature Selection

Not all imaging metrics are useful for classification because of their intrinsic redundancy. Feature selection is, therefore, important for extracting the most informative features for the specific task. Moreover, a high-dimensional data set may lead to over-fitting issues. For all these reasons, in this paper, the ReliefF feature selection algorithm was applied to each data set (see section Datasets for Machine Learning) prior to running any ML algorithm (Kononenko et al., 1997). This procedure produced a ranking of features according to their relevance in determining the class value of the data set records. Indeed, this step allowed us to select only the most informative features (and to discard the irrelevant ones) in order to improve the ML algorithms' performances. After the feature-selection stage, the ML algorithms identified as promising for the task of the study were used to construct the classification models. More details about how ReliefF was applied in our analyses have been fully reported in section Cross Validated Accuracy.

### Machine Learning Analysis

Three supervised ML approaches were considered for binary classification: artificial neural network (ANN), support vector machine (SVM), and adaptive neuro-fuzzy inference system (ANFIS). All the methods were implemented in Matlab as part of a dedicated image-based tool. The work was organized into two main steps as follows.

The selected ML algorithms (i.e., classifiers) were first run separately to

– *AIM 1* (model construction and validation): For each ML algorithm, a tuning of the relevant parameters was performed in order to identify the optimal setting to maximize the algorithm classification performance. We then proceeded by training the model and testing it independently using a balanced cross-validation approach (see section Cross Validated Accuracy). For each algorithm, for each data set (as in section Datasets for Machine Learning), we identified the best feature set to discriminate AD from VD as the one associated with the best classification performance.

Finally, we selected only the ML algorithm with the best classification performance in the binary task and we used it to

– *AIM 2* (prediction): We predicted the prevalent underlying disease in the MXD subjects, using as input the discriminant feature pattern previously identified by the selected algorithm during the training step.

#### Artificial Neural Network (ANN)

ANN are a family of learning methods inspired by biological neural networks (Haykin, 1998). In this study, two different ANN models have been implemented: multilayer perceptron (MLP) (Rumelhart et al., 1986; Rumelhart and McClelland, 1987; Geva and Sitte, 1992) and radial basis function network (RBFN) (Acosta, 1995; Bishop, 2006).

*Multilayer perceptron* (*MLP*): The MLP implementation for this study was performed in Matlab and was composed of three layers: an input layer with *n* nodes corresponding to the *n* input features from the calculated data set, a hidden layer with eight nodes and a one-node output layer. The output node resulted in either zero or one, respectively, for the AD or VD class. A sigmoidal activation function (*tansig*) was used to transform data between the input and the hidden layer as well as between the hidden layer and the output layer. A Bayesian regularization back-propagation approach was used to train the MLP network (Bishop, 2006).

*Radial Basis Function network* (*RBFN*): This is a variant of the three-layer feed-forward neural network, which uses radial basis (Gaussian) functions as its activation functions (Bishop, 2006). In this study, the RBFN was implemented in Matlab using the *newrbe* function with the spread constant for the radial basis layer set equal to 0.1.

#### Support Vector Machine (SVM)

SVM uses training data to find the maximal margin hyperplane that best divides data belonging to different groups or classes (Cortes and Vapnik, 1995). The separating hyperplane is selected to have the largest distance from the nearest training data points of any class. In the case of non-linearly separable data, a non-linear kernel function is used to project them into a higher dimensional space where they can be linearly separated. For the present study, two SVM architectures with a different non-linear kernel function were used: SVM with a radial basis function (RBF) kernel (SVM_RBF_) and SVM with an MLP sigmoid-like kernel (SVM_MLP_). For each SVM architecture, an iterative grid search was performed in order to find the optimal combination of C, σ (for SVM_RBF_), α, and *c* (for SVM_MLP_) to obtain the best SVM performance with our data.

#### Adaptive Neuro-Fuzzy Inference System (ANFIS)

ANFIS is a class of ANN that represents a trade-off between ANN and fuzzy logic systems, offering smoothness due to the fuzzy control interpolation and adaptability due to the ANN back-propagation (Zadeh, 1978). ANFIS incorporates both ANN and fuzzy logic principles and converges the benefits from both the methods in a single implementation. For this work, the ANFIS algorithm has been used as implemented in the Fuzzy Logic toolbox in Matlab using a Sugeno-type fuzzy inference system (FIS) and Gaussian functions as membership functions to specify the fuzzy sets. A hybrid learning algorithm, obtained by combining the least-squares and back-propagation gradient descent methods, was used to model the training data. For the purposes of the study, ANFIS was run using 100 epochs.

The ML models implemented for the present study can be considered as variants of the analog deep learning algorithms, which differ from the ML ones for their ability to learn features automatically at multiple levels, therefore allowing the system to learn complex functions mapping the input to the output directly from data (Goodfellow et al., 2016).

### Cross-Validated Accuracy

To improve the classification performance robustness of each ML method, for *AIM1*, we adopted a balanced Monte Carlo 10-fold cross-validation (CV) approach using 100 bootstraps. Indeed, at each iteration, the CV algorithm divided the original input data into 10 parts with the two classes (AD and VD) equally represented. This operation resulted in the creation of 100 new different CV data sets. Specifically, for each CV data set, for each ML algorithm, nine parts (i.e., 9-folds) were used as training subset, and the remaining one part (i.e., 1-fold) was used as testing subset. Moreover, the ReliefF feature-selection algorithm described above (see section Feature Selection) was here applied to each generated CV data set. For each classifier, we considered the best classification performance, obtained over the 100 bootstraps, and its related model and associated selected features as final results. This approach allowed reduction variance of the data, therefore reducing the chance of over-fitting or bias errors.

### Performance Comparison

In this study, an AD subject effectively classified as AD was considered a true positive (TP), and a VD subject effectively classified as VD was counted as a true negative (TN). An AD subject classified as VD was considered a false negative (FN), and a VD subject classified as AD was counted as a false positive (FP). Given that, for each experiment, the classification performance of the constructed model, varying the algorithm parameters as well as the number of input features presented to it, was assessed by calculating the *classification accuracy* (ACC) = (TP + TN)/(TP + TN + FP + FN), which denotes the probability of a correct classification; *sensitivity* (SEN) = TP/(TP + FN), which scores the ability of the model to detect a subject with a specific disease in a population with more than one disease; *specificity* (SPE) = TN/(TN + FP), which scores the ability of the model to correctly rule out the disease in a disease-free population; *precision* (PREC) = TP/(TP + FP), which defines the proportion of positive predictions; and *negative predictive value* (NPV) = TN/(TN + FN), which denotes the proportion of negative predictions (Bishop, 2006). For SVMs, the mean ratio of support vectors (SVr), calculated as the number of support vectors divided by the number of training subjects, was also reported as a measure of complexity degree of the models. The receiver operating characteristic (ROC) curve was calculated for each implemented model, and its area under the curve (AUC) was used to compare the different classifiers' performance (Hanley and McNeil, 1982). For each performance score, the 95% confidence intervals (95% IC) were computed using the Wilson score interval with the continuity correction approach (Newcombe, 1998).

### Prediction on MXD

The ML model that showed the highest classification performance in AIM1 was then considered to fulfill the prediction purposes of AIM2. To achieve this task, we considered the AD–VD discriminant feature pattern that resulted from the best performant classifier in AIM1. We then used this feature pattern as input for the selected ML algorithm to predict the prevalent underlying disease (i.e., AD or VD) in the MXD subjects. Finally, for each MXD subject, the predicted class from the ML algorithm on baseline MRI data was compared with the patient' 3-year follow-up clinical evaluation in order to assess the reliability of the ML predictions as well as the potential of ML to notify earlier (than clinical evidence) about the typology of the patient's dementia.

### Non-imaging Statistics

Statistical analyses were carried out using SPSS (version 21.0; SPSS, Chicago, IL, USA). Demographic, behavioral, and radiological continuous data were first tested for normality using the Shapiro–Wilk test, and differences between groups were assessed with different tests depending on the typology of the variables (binary, normally or non-normally distributed). Chi-square tests were performed to compare frequency distributions of gender in the three groups. One-way analysis of variance (ANOVA) with Bonferroni correction was used to assess whether age was statistically different between groups (AD, VD, and MXD). A non-parametric Kruskal–Wallis test was applied to test differences between the groups in education level, clinical indices (HS, Fazekas and AWMRC, see section Clinical and Neuropsychological Assessment for details), and neuropsychological scores (attention, memory, language, executive and visuospatial cognitive domains). A non-parametric Mann–Whitney *U*-test was performed to test differences between paired groups in HS and Fazekas. A further Mann–Whitney *U*-test was applied to test differences in the features that the most performant ML algorithm identified as relevant to separate AD and VD.

## Results

### Clinical Findings

The demographic and clinical characteristics of patients are summarized in Table 2. Significant differences were found in gender between AD and VD as well as between VD and MXD groups. Fazekas and HS scores showed significant differences between AD and VD. Significant differences were also found in HS when comparing MXD vs. AD and VD groups. Fazekas scores were also significantly higher in MXD compared to AD.

**Table 2:** Demographics of AD, VD, and MXD groups.

	**AD**	**VD**	**MXD**	***p***
N	33	27	15	
Sex (M/F)	15/18	3 /24^†^	7 /8^†^	<0.05^*^
Age	72.88 ± 7.31	76.67 ± 7.77	76.33 ± 6.78	0.123
Education (years)	6.47 ± 3.26	5.52 ± 2.13	5.08 ± 1.65	0.363
MMSE	16.10 ± 6.32	17.89 ± 4.15	18.59 ± 4.17	0.245
Memory	0.65 ± 0.74	0.73 ± 0.61	0.64 ± 0.70	0.832
Attention	0.91 ± 0.90	0.71 ± 0.68	0.48 ± 0.53	0.185
Language	1.06 ± 1.26	1.02 ± 1.06	1.08 ± 1.48	0.986
Executive function	0.60 ± 0.91	0.45 ± 0.76	0.38 ± 0.87	0.665
Visuospatial skills	0.66 ± 1.41	0.52 ± 1.19	0.30 ± 1.25	0.682
Hachinski score (HS)	2.97 ± 0.84^†^	8.27 ± 1.58^†^	5.80 ± 2.11^†^	<0.001^*^
Fazekas score	2.65 ± 1.27^†^	4.63 ± 1.52^†^	4.21 ± 1.82^†^	<0.001^*^

*Demographic and clinical scores for Alzheimer's disease (AD), vascular dementia (VaD), and mixed VD-AD dementia (MXD) groups. Values are expressed as mean ± SD. MMSE, Mini Mental State Examination. p-values show statistically significant differences between AD, VD and MXD groups*.

* *p < 0.05 between AD and VD*.

† *p < 0.05 between MXD and AD or VD or between AD and VD. Statistically significant p-values have been highlighted in bold*.

### Classification Results

**Figure 2:**
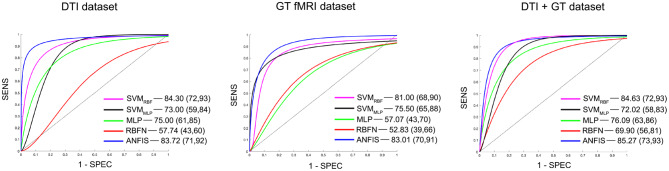
Details of the ROC curves and relative AUC (95% IC) values obtained from each run classifier (SVM_RBF_, SVM_MLP_, MLP, RBFN, and ANFIS) using input data from the DTI data set (on the **left**), the GT fMRI data set (in the **middle**), and the DTI + GT data set (on the **right**).

Three different kinds of ML algorithms (SVM, ANN, and ANFIS) were used to identify the best feature pattern to classify AD from VD. The analyses yielded the following results (details of the classification performances of each classifier on the three data sets have been fully reported in Table 3, Figure 2 and Table S2).

**Table 3:** Classification performance scores obtained using data, respectively, from the DTI, GT fMRI, and DTI + GT data sets.

	**ACC (%)**	**SENS (%)**	**SPEC (%)**	**PREC (%)**	**NPV (%)**
**DTI DATASET**					
SVM_RBF_	79.75 (66,89)	68.00 (54,79)	91.50 (80,97)	88.89 (76,95)	88.89 (77,95)
SVM_MLP_	73.00 (59,84)	63.00 (49,75)	83.00 (70,92)	78.75 (65,88)	79.00 (65,88)
MLP	75.00 (61,85)	74.00 (60,85)	76.00 (62,86)	75.51 (62,86)	74.50 (60,85)
RBFN	60.25 (46,73)	65.00 (51,77)	55.50 (42,69)	59.36 (45,72)	38.34 (25,52)
ANFIS	**83.50 (71,92)**	**81.00 (68,90)**	**86.00 (74,94)**	**85.26 (73,93)**	**79.90 (66,89)**
**GT fMRI DATASET**					
SVM_RBF_	81.00 (68-90)	93.50 (83,99)	68.50 (55,80)	74.80 (61,85)	74.80 (61,85)
SVM_MLP_	78.25 (64-88)	81.00 (68,90)	75.50 (62,86)	76.78 (63,86)	76.78 (63,87)
MLP	58.25 (44,71)	55.50 (42,69)	61.00 (47,74)	58.73 (45,72)	57.81 (43,70)
RBFN	55.75 (42,69)	55.50 (42,69)	56.00 (42,69)	55.78 (42,69)	17.06 (8,29)
ANFIS	**82.75 (69,91)**	**73.50 (64,88)**	**92.00 (81,97)**	**90.18 (78,96)**	**73.60 (59,84)**
**DTI+GT DATASET**					
SVM_RBF_	84.75 (72,93)	84.00 (71,92)	85.50 (73,93)	85.28 (73,93)	85.28 (72,93)
SVM_MLP_	74.75 (61,85	65.00 (51,77)	84.50 (72,93)	80.75 (68,90)	80.75 (67,89)
MLP	76.75 (63,87)	74.00 (60,85)	79.50 (66,89)	78.31 (65,88)	75.35 (61.85)
RBFN	62.75 (49,75)	86.50 (74,94)	39.00 (27,53)	58.64 (45,71)	74.28 (60,84)
ANFIS	**85.25 (73,93)**	**82.00 (69,91)**	**88.50 (77,96)**	**87.70 (76,95)**	**81.69 (68,90)**

*Each performance score is expressed as a percentage (%). For each score, the 95% IC is also reported in brackets. Classification performance scores (accuracy, sensitivity, specificity and AUC) for pairwise classifiers are expressed as percentage (%) with 95% confidence intervals in brackets. AUC, Area under receiver operating characteristic curve. ANFIS emerged as the most performant method in discriminating at baseline AD from VD independently of the data set (scores highlighted in bold)*.

*Classification using the DTI data set:* ANFIS showed the best classification performance in dividing AD from VD subjects, reaching a classification accuracy (ACC) of 83.50% and area under the ROC curve (AUC) equal to 83.72% (see Table 3 and Figure 2), using a feature pattern including only four FA features from four brain regions, including the left hippocampus and three areas of the corpus callosum (body anterior, genu, and splenium, see Table S2). SVM_RBF_ (C = 1, σ = 1.87, SVr = 78.57%) also discriminated the two patient groups with a relatively high ACC = 79.75% and AUC = 84.30%, using a feature pattern of seven FA features from seven distinct brain regions including both hippocampi, left cingulum, left thalamus, and the corpus callosum (body anterior/posterior, genu, see Table S2). The remaining ML algorithms (SVM_MLP_, MLP, and RBFN) resulted instead in lower classification performance (ACC ≤ 75%) as reported in Table 3.

*Classification using the GT fMRI data set:* ANFIS reached the highest performance score with ACC = 82.75%, AUC = 83.01% using 10 features (mainly DEG and Eloc_norm_) from nine distinct brain areas, including bilateral superior parietal gyrus, right anterior cingulum, left precuneus and cuneus, left superior frontal gyrus, right postcentral gyrus, and bilateral fusiform gyri (Table 3, Figure 2 and Table S2). SVM_RBF_ (C = 0.14, σ = 1.73, SVr = 98.21%) resulted in a relatively lower (than ANFIS) performance, scoring ACC = 81% and AUC = 81% using six features (mainly DEG and Eloc_norm_) from six distinct brain regions, including left precuneus and cuneus, right middle and left superior frontal gyri, right postcentral gyrus, and right fusiform gyrus (Table S2). Moreover, SVM_MLP_ (C = 0.37, α = 1, *c* = −1, SVr = 80.36%) showed a classification performance closer (even lower) to those of ANFIS and SVM_RBF_, reaching ACC = 78.25% and AUC = 75.50% (Table 3 and Figure 2) using only three features (DEG and Eglob) from two distinct brain areas: right fusiform and superior parietal gyri (Table S2). The remaining ANN algorithms (MLP and RBFN) resulted, instead, in poorer classification performance (ACC < 60%) as reported in Table 3.

*Classification using the DTI* + *GT data set:* Even when using the multimodal data set (DTI + GT), ANFIS showed the best performance (Table 3 and Figure 2), scoring ACC = 85.25% and AUC = 85.27% using a total of 10 features, including five FA features from DTI involving left hippocampus, left thalamus, and corpus callosum (genu, body anterior/posterior) and five features from GT fMRI, involving right anterior cingulum, right superior parietal gyrus, left precuneus, and bilateral fusiform gyri (see Table 4 and Table S2). After ANFIS, SVM_RBF_ (C = 2.72, σ = 2.12, SVr = 91.07%) resulted in the second most performant classifier, reaching ACC = 84.75% and AUC = 84.63%, using a total of nine features, including four FA features from DTI involving left hippocampus, left thalamus, and corpus callosum (genu, body anterior); and five features from GT fMRI, involving right anterior cingulum, right superior parietal gyrus, left precuneus, left cuneus, and right precentral gyrus (Table S2). The remaining algorithms (SVM_MLP_, MLP, and RBFN) showed better results when compared to the scores obtained using the unimodal data sets but lower (ACC < 80%) than ANFIS performance overall (see Table 3).

**Table 4:** Details (number of features, brain area, and MRI metric) of the discriminant feature pattern identified by ANFIS, which resulted the best classifier in separating AD and VD using data from the multimodal data set (i.e., DTI + GT data set).

	**ACC (%)**	**N features**	**Area**	**MRI metric**	**ReliefF ranking score**
ANFIS	85.25	10	L Thalamus	FA	0.384
			Corpus callosum body anterior	FA	0.312
			R Anterior Cingulum	DEG	0.299
			Corpus callosum genu	FA	0.284
			L Precuneus	DEG	0.268
			L Hippocampus	FA	0.255
			R Superior Parietal gyrus	DEG	0.206
			L Fusiform gyrus	DEG	0.202
			Corpus callosum body posterior	FA	0.172
			R Fusiform gyrus	DEG	0.149

*The listed features are reported reflecting the ranking order given by ReliefF (i.e., top feature corresponds to the most informative one). For each feature, the ReliefF ranking score has also been added. L, left part; R, right part 2*.

### Prediction Results

**Figure 3:**
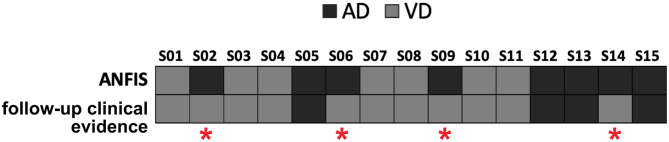
Predictions of the prevalent underlying disease (*dark gray* squares for AD, *light gray squares* for VD) on the MXD subjects performed by ANFIS using the feature pattern reported in Table 4. ANFIS correctly predicted the class for 11 out of the 15 MXD subjects (77.33% correct prediction rate). A red asterisk highlights the four subjects for whom ANFIS predicted a class that was in discordance with the clinical evidence at follow-up.

ANFIS resulted in the most performant classifier in separating AD from VD subjects (see Table 3), and its performance was maximized when using feature patterns that included multimodal features (i.e., combined DTI and GT features; see Table 4). Therefore, ANFIS was chosen to perform the subsequent analysis (i.e., AIM2 analysis). To achieve our AIM2 goal (prediction), the discriminant 10-feature pattern identified at AIM1 (see Table 4) was used as input to ANFIS to make predictions on the prevalent underlying disease (i.e., AD or VD) in MXD subjects. According to the evidence of patients' diagnosis from clinical evaluation at 3-year follow-up, ANFIS correctly predicted the prevalent underlying disease for 11 of the 15 MXD subjects, which means a 77.33% correct prediction rate (see Figure 3).

## Discussion

In this paper, ML algorithms were combined with advanced qMRI metrics to assess their potential to automatically discriminate AD from VD. First, quantitative metrics from DTI (FA and MD) and rs-fMRI (GT metrics) were extracted and used to build two unimodal data sets (DTI data set, GT fMRI data set). Then, the two data sets were unified in order to obtain a multimodal structural-functional data set (DTI + GT data set). Multiple supervised ML algorithms were applied on each data set, and classification results were obtained. Various ML methods have been used in literature to identify dementia-like diseases, including SVM with different kernels and ANNs, such as RBFN, MLP, and ANFIS. We examined these classifiers to find the most performant for our study. Finally, the best discriminant feature pool was identified and used as input for the most performant classifier to predict the prevalent underlying disease (i.e., AD rather than VD) on a group of subjects whose diagnosis, based on clinical evaluation, was unclear and defined as mixed between VD and AD (MXD group). The algorithm's accuracy in predicting the MXD prevalent underlying disease was compared with the diagnosis evidence obtained from 3-year follow-up clinical screening.

Among the evaluated algorithms, ANFIS emerged as the most performant method in discriminating at baseline AD from VD independently of the data set (unimodal or multimodal) used as input (see Table 3). Indeed, ANFIS achieved the highest classification accuracy (ACC > 84%) when using the multimodal data set as input, i.e., when providing both structural and functional information as input, simultaneously. These results are in line with the findings of a number of previous studies that investigated the use of multimodal data to automatically diagnose AD (and MCI) from healthy subjects (Zhang et al., 2011; Liu F. et al., 2014; Liu M. et al., 2014; Lei et al., 2016; Liu et al., 2018). All these studies concluded that unimodal data generally provides incomplete information to accurately diagnose dementia, and multimodal data tends to boost the classification accuracy due to the complementary information.

**Figure 4:**
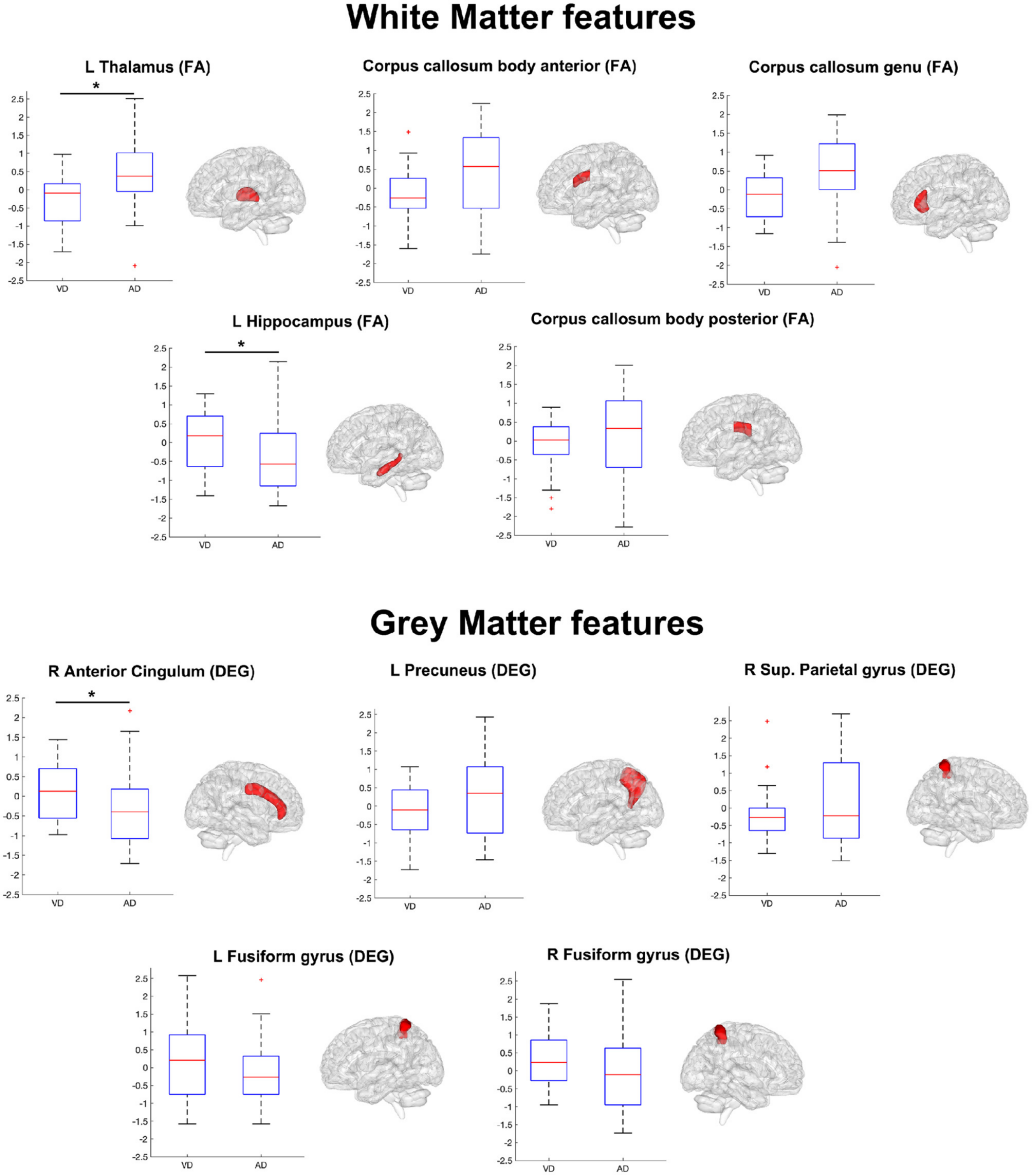
Boxplots representing the summary of the 10 features (WM features on top, GM features on bottom) in AD and VD groups. The ensemble of these features (see also Table 4) formed the discriminant pattern that was also used to predict the prevalent underlying disease in MXD subjects. Each feature has been tested with the Mann–Whitney *U* test in order to assess significant differences between AD and VD values. An asterisk mark has been added on the top of the boxplot of the features with values significantly (*p* < 0.05) different between the AD and VD populations.

Table 4 lists the pattern of features that ANFIS identified as the most discriminant to separate AD from VD. Brain areas such as the thalamus, hippocampus, precuneus, and anterior cingulum were identified as relevant to discriminate AD and VD subjects. Because AD dementia involves a significant deficit in the anterograde episodic memory, areas such as the hippocampus and the thalamus were expected to come out as relevant for the classification problem (Jahn, 2013; Aggleton et al., 2016). The anterior cingulum and the precuneus are both relevant for episodic memory as well and are also core regions of the default mode network (DMN), which has been extensively studied because of its severe alterations in AD (Greicius et al., 2004; Castellazzi et al., 2014). According to the implemented feature selection algorithm (ReliefF), the FA of the left thalamic white matter resulted as the most informative feature to discriminate AD from VD (Table 4). The thalamus region is involved in neural networks that sustain complex cognitive and behavioral functions, and a link has been demonstrated between thalamic dysfunctions and episodic memory impairment in AD (Aggleton et al., 2016). Indeed, our results revealed that the left thalamic FA was significantly higher (*p* < 0.05) in AD compared to VD (see Figure 4), therefore suggesting that, in AD, the thalamic tracts might be more coherent possibly due to a loss of crossing fibers (Teipel et al., 2014). Moreover, the anterior cingulum nodal degree (DEG), which reflects the number of functional connections between ACC and other parcellated brain areas, also resulted as an informative feature to discriminate AD from VD (Table 4). Indeed, the AD group showed significantly (*p* < 0.05) lower DEG scores than VD (see Figure 4) in the anterior cingulum, therefore suggesting a more severe disconnection of this area in AD patients. In our study, the left hippocampal white matter FA, extracted by the TBSS analysis (Palesi et al., 2018), emerged as a relevant feature to separate AD from VD subjects (Table 4). Indeed, the FA in the left hippocampal area resulted significantly (*p* < 0.05) reduced in AD compared to VD (see Figure 4). This result may be interpreted as the evidence of more severe tract disconnection affecting the WM portion of the left hippocampus in AD.

When using the AD–VD discriminant feature pattern (Table 4) as input to ANFIS to predict the prevalent underlying disease in MXD patients starting from their baseline data, the ML algorithm achieved a correct prediction rate of 77.33% (Figure 3). The accuracy of ANFIS predictions has been validated against the diagnosis obtained from MXD patients' clinical screening at 3-year follow-up. The high matching rate between ANFIS predictions and clinical follow-up suggests the potential of the ML approach combined with MRI-derived indexes to obtain an accurate detection of the prevalent disease in individual subjects with MXD 3 years prior to clinical evidence. Considering that novel therapeutic approaches to treat the AD condition, such as those using monoclonal antibodies, are more effective at early stages of the pathology (van Dyck, 2018), the ML-based prediction of AD in subjects clinically diagnosed with MXD could be crucial in order to promptly administrate these emerging treatments.

From the point of view of study design, the present work is a cross-sectional investigation, and although the ML algorithms responded with high performance in discriminating clinical AD vs. VD, their implication for MXD prognosis and their integration in patients' management will require appropriate longitudinal data. From a technical perspective, this study used ML to disentangle the feature pattern that better identifies the AD profile and separate it from the VD one, suggesting a possible solution to identify the most likely disease progression in subjects with MXD. Indeed, this will help to identify the most suitable and prompt therapy for each MXD subject.

A different approach for classification, which is becoming very popular, is to use deep learning algorithms, which differ from ML for their ability to learn features automatically at multiple levels, therefore allowing the system to learn complex functions mapping the input to the output directly from data (Goodfellow et al., 2016; Qureshi et al., 2019). It could be envisaged that ML and deep learning methods could lead beyond current clinical diagnosis by establishing, in an unsupervised fashion, groups of patients with similar MRI and clinical scores. This may lead beyond current clinical classification of dementia and require a major clinical effort to understand the biological mechanisms that differentiate potentially novel disease patterns, but this is beyond the scope of this study.

In this study, we used the AAL atlas to parcellate the brain before applying GT. The AAL atlas parcels the brain on the anatomical traits, which do not exactly match functional brain organization, and this may degrade performance metrics. Indeed, Shirer et al. (2012) showed that an atlas based on functional (rather than structural) ROIs provides better classification performances for the analysis of fMRI data. Nonetheless, there is still no consensus about the optimal strategy for brain parcellation (Arslan et al., 2018).

A final consideration is that the 85% accuracy of ANFIS was achieved solely based on qMRI features without including clinical tests. This means that objective qMRI features are able to perform the AD vs. VD classification alone, suggesting that this ML approach provides a substantial contribution for diagnosis. Future works should explore the pattern of features identified here together with clinical and neuropsychological variables and metrics from biological samples to improve the accuracy of the algorithm even further.

## Conclusions

This study, which combines local DTI metrics and GT measures from rs-fMRI data with ML, shows great potential for the automatic classification of AD and VD in patients with mixed clinical assessment. Indeed, multimodal features from MRI could be used to automatically separate AD from VD patients with high accuracy and balanced sensitivity and specificity. Among the pool of ML algorithms available to the user, ANFIS appeared to overcome others in classification performance. Results were consistent with reported literature in identifying specific brain regions such as the thalamus, hippocampus, and anterior cingulum with specific dementia types. Interestingly, our analytical method, by using baseline data, provided early prediction of disease type (AD or VD) in patients with clinical mixed dementia symptoms. Considering these encouraging results, we strongly believe that ML coupled to high-resolution MRI will provide a suitable approach to support clinicians in their clinical work, helping them to improve their diagnostic and prognostic accuracy as well as therapy and patient management.

## Data Availability Statement

The datasets generated for this study are available on request to the corresponding author.

## Ethics Statement

The studies involving human participants were reviewed and approved by Comitato Etico Pavia, Fondazione IRCCS Policlinico San Matteo, Pavia, Italy. The patients/participants provided their written informed consent to participate in this study.

## Author Contributions

GC, MGC, DM, GMa, and CG conceptualized the study. GC designed and performed the analyses with support AR and FP. PV and NA acquired all MRI data. MCR, SB, FD, ES, AC, and GMi enrolled patients and acquired all the non-imaging data with the help of PV and NA. GMa, CG, DM, and MCR provided support and guidance with data interpretation with the clinical contribution of all physicians. GC, ED'A, GMa, and CG wrote the manuscript, with comments from all other authors.

## Conflict of Interest

The authors declare that this work was supported by the Italian Ministry of Health (RC2016) for data collection. CG received research funding from EPSRC, Horizon2020, Biogen Idec, Novartis, Wings for Life. These funders were not involved in the study design, collection, analysis, interpretation of data, the writing of this article or the decision to submit it for publication. Moreover, this study was also funded by the UK MS Society (programme grant number 984) and the ECTRIMS Postdoctoral Research Fellowship Programme to GC. The UK MS Society and ECTRIMS were not involved in the study design, collection, analysis, interpretation of data, the writing of this article or the decision to submit it for publication.
