# Supplementary material for: A Machine Learning Approach for the Differential Diagnosis of Alzheimer and Vascular Dementia Fed by MRI Selected Features
Source: Front Neuroinform. 2020 Jun 11;14:25. doi: 10.3389/fninf.2020.00025 (PMC7300291; doi:10.3389/fninf.2020.00025)
Supplement: Supplementary file 1 [file Table_1.PDF]

Supplementary Material

Table S1

List of brain regions extracted from the AAL template and their abbreviations as used in this study.

Index	Regions	Abbreviation
(1,2)	Precental gyrus	PreCG
(3,4)	Superior frontal gyrus, dorsolateral	SFGdor
(5,6)	Superior frontal gyrus, orbital part	ORBsup
(7,8)	Middle frontal gyrus	MFG
(9,10)	Middle frontal gyrus, orbital part	ORBmid
(11,12)	Inferior frontal gyrus, opercular part	IFGoperc
(13,14)	Inferior frontal gyrus, triangular part	IFGtriang
(15,16)	Inferior frontal gyrus, orbital part	ORBinf
(17,18)	Rolandic operculum	ROL
(19,20)	Supplementary motor area	SMA
(21,22)	Olfactory cortex	OLF
(23,24)	Superior frontal gyrus, medial	SFGmed
(25,26)	Superior frontal gyrus, medial orbital	ORBsupmed
(27,28)	Gyrus rectus	REC
(29,30)	Insula	INS
(31,32)	Anterior cingulate and paracingulate gyri	ACG
(33,34)	Median cingulate and paracingulate gyri	MCG
(35,36)	Posterior cingulate gyrus	PCG
(37,38)	Hippocampus	HIP
(39,40)	Parahippocampal gyrus	PHG
(41,42)	Amygdala	AMYG
(43,44)	Calcarine fissure and surrounding cortex	CAL
(45,46)	Cuneus	CUN
(47,48)	Lingual gyrus	LING
(49,50)	Superior occipital gyrus	SOG
(51,52)	Middle occipital gyrus	MOG
(53,54)	Inferior occipital gyrus	IOG
(55,56)	Fusiform gyrus	FFG
(57,58)	Postcentral gyrus	PoCG
(59,60)	Superior parietal gyrus	SPG
(61,62)	Inferior parietal, but supramarginal and angular gyri	IPL
(63,64)	Supramarginal gyrus	SMG
(65,66)	Angular gyrus	ANG
(67,68)	Precuneus	PCUN
(69,70)	Paracentral lobule	PCL
(71,72)	Caudate nucleus	CAU
(73,74)	Lenticular nucleus putamen	PUT
(75,76)	Lenticular nucleus, pallidum	PAL
(77,78)	Thalamus	THA
(79,80)	Heschl gyrus	HES
(81,82)	Superior temporal gyrus	STG
(83,84)	Temporal pole: superior temporal gyrus	TPOsup
(85,86)	Middle temporal gyrus	MTG
(87,88)	Temporal pole: middle temporal gyrus	TPOmid
(89,90)	Inferior temporal gyrus	ITG
(91,92)	Cerebelum_Crus I	CRBLCrus1
(93,94)	Cerebelum_Crus II	CRBLCrus2
(95,96)	Cerebelum lobule III	CRBL3

(97,98)	Cerebelum lobule IV-V	CRBL45
(99,100)	Cerebelum lobule VI	CRBL6
(101,102)	Cerebelum lobule VIIb	CRBL7B
(103,104)	Cerebelum lobule VIII	CRBL8
(105,106)	Cerebelum_lobule IX	CRBL9
(107,108)	Cerebelum lobule X	CRBL10
(109)	Vermis I-II	Vermis12
(110)	Vermis III	Vermis3
(111)	Vermis IV-V	Vermis45
(112)	Vermis VI	Vermis6
(113)	Vermis VII	Vermis7
(114)	Vermis VIII	Vermis8
(115)	Vermis IX	Vermis9
(116)	Vermis X	Vermis10

Table S2

Feature patterns relative to the best performance obtained by each classifier depending on the dataset considered (DTI dataset, fMRI GT dataset or DTI + GT dataset). For each classifier, the list of the areas and relative metrics reflect the ReliefF ranking order (i.e. the first area is the most relevant for the classification task according to ReliefF).

	ACC (%)	N features	Area (ordered according to ReliefF ranking)	MRI metric
DTI dataset				
SVM_{RBF}	79.75	7	Corpus callosum genu	FA
			L Thalamus	FA
			Corpus callosum body anterior	FA
			L Hippocampus	FA
			R Hippocampus	FA
			L Cingulum	FA
			Corpus callosum body posterior	FA
SVM_{MLP}	73.00	3	L Hippocampus	FA
			Corpus callosum genu	FA
			L Thalamus	FA
MLP	75.00	2	L Thalamus	FA
			L Hippocampus	FA
RBFN	60.25	1	L Thalamus	FA
ANFIS	83.50	4	Corpus callosum genu	FA
			Corpus callosum body anterior	FA
			Corpus callosum splenium	FA
			L Hippocampus	FA
fMRI GT dataset				

SVM_{RBF}	81.00	6	L Precuneus	DEG
			L Cuneus	DEG
			L Superior Frontal gyrus	$Eloc_{norm}$
			R Fusiform gyrus	DEG
			R Postcentral gyrus	$Eloc_{norm}$
			R Middle Frontal gyrus	$Eloc_{norm}$
SVM_{MLP}	78.25	3	Global efficiency	Eglob
			R Fusiform gyrus	DEG
			R Superior Parietal gyrus	DEG
MLP	58.25	1	L Precuneus	DEG
RBFN	55.75	1	L Superior Frontal gyrus	$Eloc_{norm}$
ANFIS	85.75	10	R Superior Parietal gyrus	DEG
			R Anterior Cingulate gyrus	DEG
			L Precuneus	DEG
			R Fusiform gyrus	DEG
			L Cuneus	DEG
			L Superior Parietal gyrus	DEG
			L Superior Frontal gyrus	$Eloc_{norm}$
			Global efficiency	Eglob
			R Postcentral gyrus	$Eloc_{norm}$
			L Fusiform gyrus	DEG
DTI+ GT dataset				
SVM_{RBF}	84.75	9	R Anterior Cingulum	DEG
			L Hippocampus	FA
			Corpus callosum genu	FA
			L Thalamus	FA
			R Superior Parietal gyrus	DEG
			Corpus callosum body anterior	FA
			L Precuneus	DEG
			R Precentral gyrus	*CC*
			L Cuneus	*CC*
SVM_{MLP}	74.75	4	Corpus callosum genu	FA
			L Thalamus	FA
			Global efficiency	Eglob
			R Superior Parietal gyrus	DEG
MLP	76.75	2	L Thalamus	FA
			L Hippocampus	FA
RBFN	62.75	2	Graph average CC	Cm
			L Thalamus	FA
ANFIS	85.25	10	L Thalamus	DEG
			Corpus callosum body anterior	FA
			R Anterior Cingulate gyrus	DEG
			Corpus callosum genu	FA
			L Precuneus	DEG
			L Hippocampus	FA
			R Superior Parietal gyrus	DEG
			L Fusiform gyrus	DEG
			Corpus callosum body posterior	FA
			R Fusiform gyrus	DEG
